# Humoral and cellular response to the third COVID-19 vaccination in patients with inborn errors of immunity or mannose-binding lectin deficiency: A prospective controlled open-label trial

**DOI:** 10.1007/s00508-024-02459-6

**Published:** 2024-10-24

**Authors:** Matthias G. Vossen, Felix Kartnig, Daniel Mrak, Elisabeth Simader, Karin Stiasny, Renate Kain, Thomas Perkmann, Helmuth Haslacher, Judith H. Aberle, Leonhard X. Heinz, Daniela Sieghart, Heinz Burgmann, Daniel Aletaha, Clemens Scheinecker, Michael Bonelli, Lisa Göschl

**Affiliations:** 1 Division of Infectious Diseases and Tropical Medicine, Department of Medicine I, Medical University of Vienna, Vienna, Austria; 2 Division of Rheumatology, Department of Internal Medicine III, Medical University of Vienna, Währinger Gürtel 18–20, 1090 Vienna, Austria; 3 Center for Virology, Medical University of Vienna, Vienna, Austria; 4 Medical University of Vienna, Clinical Institute for Pathology, Vienna, Austria; 5 Clinical Institute for Laboratory Medicine, Medical University of Vienna, Vienna, Austria; 6 Comprehensive Center for Infection Medicine, Medical University of Vienna, Austria (CCIM), Vienna, Austria; 7 Comprehensive Center for Inflammation and Immunity, Medical University of Vienna, Austria (CCII), Vienna, Austria

**Keywords:** Hypogammaglobulinemia, Immunosuppression, Common variable immunodeficiency, Primary immunodeficiency disorder, Mannose-binding lectin

## Abstract

Impaired immune response to COVID-19 (coronavirus disease 2019) vaccination has been reported in patients with inborn errors of immunity (IEI). Repetitive vaccinations are recommended for this vulnerable group. Due to the high diversity within IEI patients, additional safety and immunogenicity data are needed to better understand these aspects especially in less common immunodeficiency syndromes. In this prospective open-label clinical trial, we assessed the humoral immune response and the T‑cell response in patients with IEI or severe MBL (mannose-binding lectin) deficiency (IEI/MBLdef) after three vaccinations. A total of 16 patients and 16 matched healthy controls (HC) with suboptimal humoral response defined by anti-SARS-CoV‑2 RBD (severe acute respiratory syndrome coronavirus type 2 receptor binding domain) antibodies below 1500 BAU/ml (binding antibody units per ml) after the second COVID-19 vaccination were enrolled in this study and qualified for a third mRNA vaccine dose. After 4 weeks following vaccination, 100% of HC and 75% of IEI/MBLdef patients exhibited anti-SARS-CoV‑2 RBD antibodies > 1500 BAU/ml, although the difference was not statistically significant (75% vs. 100%; *p* = 0.109). Although post-vaccination IEI/MBLdef patients demonstrated significantly increased anti-SARS-CoV‑2 RBD antibodies and neutralizing antibodies compared to baseline, these responses were significantly lower in IEI/MBLdef patients compared to HCs. Notably, the third vaccination augmented the cellular immune response to both wild-type and omicron peptide stimulation. No serious adverse events were reported within the 4‑week follow-up period and, importantly, vaccination had little to no effect on the long-term disease activity and fatigue. This trial strongly supports the recommendation of repeated COVID-19 vaccinations for patients suffering from immunodeficiencies, especially when they exhibit an initially limited response to the vaccine.

**Supplementary Information:**

The online version of this article (10.1007/s00508-024-02459-6) contains supplementary material, which is available to authorized users

## Introduction

Despite the increasing availability of direct-acting antiviral agents and anti-SARS-CoV‑2 specific antibodies for therapeutic and prophylactic purposes, vaccination against COVID-19 remains the most effective strategy for reducing disease severity in individuals [1]. Additionally, viral immune evasion through mutation can diminish the efficacy of the humoral immune response, while the T‑cell response tends to be more robust and thus preserved across all known SARS-CoV‑2 variants, offering a level of protection for individuals with compromised humoral response [2]. Patients with inborn errors of immunity (IEI) constitute a highly diverse and continually expanding group, encompassing over 480 different monogenic mutations [3]. The IEI patients exhibited a higher prevalence of COVID-19 disease compared to the general population before the widespread deployment of vaccines [4]; however, data regarding the severity of COVID-19 in this specific cohort are conflicting [5, 6]. It has been demonstrated that IEI patients experience increased morbidity and mortality from COVID-19 compared to the general population [5, 7]. While there is strong evidence that vaccination may offer protection to these patients [8, 9], vaccine hesitancy is observed in this cohort, primarily due to concerns about post-vaccination disease flares and uncertainties regarding vaccine safety [10, 11].

There is a growing amount of data which support the safety and immunogenicity of COVID-19 vaccinations in patients with IEI or severe MBL deficiency (IEI/MBLdef) [9, 12–17] and a third dose has been explicitly recommended for immunocompromised patients [12, 18–23]. These patients exhibit heterogeneous immune responses to different vaccines based on their underlying pathology. Our trial aimed to bolster this evidence, with a particular focus on some of the less common immunodeficiency syndromes. Additionally, we aimed to identify and describe any potential triggering of autoimmune events as thoroughly as possible.

## Patients and methods

### Trial design and participants

Briefly, 16 IEI patients as well as 16 HCs (age ≥ 18 years) with an anti-SARS-CoV‑2 RBD antibody level of < 1500 BAU/ml (binding antibody units per ml) after primary COVID-19 vaccination were included. The data of the previous vaccination, immunosuppressive treatment, diagnostic criteria, autoimmune phenomena and infectious complications are summarized in Supplementary Table 1. Major exclusion criteria included allergies to vaccines and previous infection with SARS-CoV‑2, which had been defined as a positive COVID-19 PCR test. All subjects completed the trial. The trial protocol was approved by competent authorities and the ethics committee of the Medical University of Vienna (No.: 1583/2021) and was registered in the European Clinical Trials Database (EudraCT No: 2021-002693-10). Subjects gave informed consent to participate in the study before taking part. The study procedures were performed in accordance with good clinical practice guidelines and the Declaration of Helsinki.

### Procedures

During the trial five visits were performed. All participants were vaccinated using either BNT162b2 (30 µg dose) (BioNTech Manufacturing GmbH, Mainz, Germany) or mRNA-1273 (100 µg dose) (Moderna Biotech Spain, S.L. Madrid, Spain), depending on their choice. A paper-based patient diary was used to collect safety data for 7 days. The patient global assessment (PGA) of the underlying disease and fatigue was quantified using a patient global numeric rating scale (NRS) ranging from 0 to 10, where 0 indicates no disease activity or fatigue, and 10 represents the highest conceivable level of disease activity or fatigue. This assessment was conducted at week 0 and at week 4.

### Analysis of immune response

The humoral immune response and the neutralizing capability of the patient’s anti-spike protein antibodies was evaluated at week 4, as described previously [24]. The T‑cell response was assessed 1 week after immunization by enzyme-linked immunosorbent spot (ELISpot) assays, as previously detailed [24].

### Statistical analysis

Data analysis was performed in R (The R Foundation for Statistical Computing, Vienna, Austria) version 4.1.2. Fisher’s exact test was used to assess different rates of vaccine responses. Normality was tested by the Shapiro-Wilk normality test and non-parametric tests were used according to the data distribution. The Wilcoxon signed-rank test was applied to compare anti-SARS-CoV‑2 RBD antibodies, NT (neutralization test) titers and T‑cell data. Spearman’s rank correlation coefficient was used for the numerical assessment of correlations. For intergroup comparison, a two-sided Wilcoxon test was used. GraphPad Prism (GraphPad Software, Boston, MA, USA) (V.9.3.1) was used for the graphical presentation of selected the data.

## Results

### Patient characteristics

In this trial 16 adult immunocompromised patients and 16 healthy control (HCs), who had been previously vaccinated twice with a SARS-CoV‑2 vaccine and displayed SARS-CoV‑2 RBD antibody levels below 1500 BAU/ml were enrolled. Among the patient cohort, 11 patients were diagnosed according to the recent IUIS (International Union of Immunological Societies) classification [3] and 5 patients suffered from severe mannose-binding lectin (MBL) deficiency defined by MBL levels below 30 ng/ml, as described in Supplementary Table 1. Of the patients three preferred to be boosted with mRNA-1273, all other patients were immunized using BNT162b2 (Supplementary Table 1). The average patient age was 44 years (± 12 years), with a male-to-female ratio of 0.33. The sex and age-matched HC cohort included 16 adults with an average age of 44 years (± 11 years). All HCs received a third vaccination with BNT162b2. Patient and HC characteristics are summarized in Table 1.

**Table 1:** Characteristics of patients and healthy control subjects who completed week 4

		Patients	Healthy controls
*n*	–	16	16
Age (years)	–	43.6 (± 12.1)	44.2 (± 11.4)
Sex: male (%)	–	4 (25%)	4 (25%)
Primary vaccination compound	BNT162b2 (%)	13 (81.25%)	16 (100%)
	mRNA-1273 (%)	1 (6.25%)	0 (0%)
	ChAdOx1 (%)	2 (12.5%)	0 (0%)
3rd vaccination compound	BNT162b2 (%)	13 (81.25%)	16 (100%)
	mRNA-1273 (%)	3 (18.75%)	0 (0%)
Days between 2nd and 3rd vaccination	–	140 (± 31)	253 (± 35)
SARS-CoV‑2 RBD titer at screening	–	375.5 BAU/ml [70.93–850]	455 BAU/ml [253.8–702]
NT titer at screening	–	12.5 [0–20]	15 [10–18.75]

Data are presented as *n* (%), mean ± SD (age and days between 2nd and 3rd vaccination), or median (1st quartile–3rdquartile)

*NT* neutralization test, *S* spike, *WT* wild type, *RBD* receptor binding domain, *SARS-CoV‑2* severe acute respiratory syndrome coronavirus type 2

### Humoral immune response

**Fig. 1:**
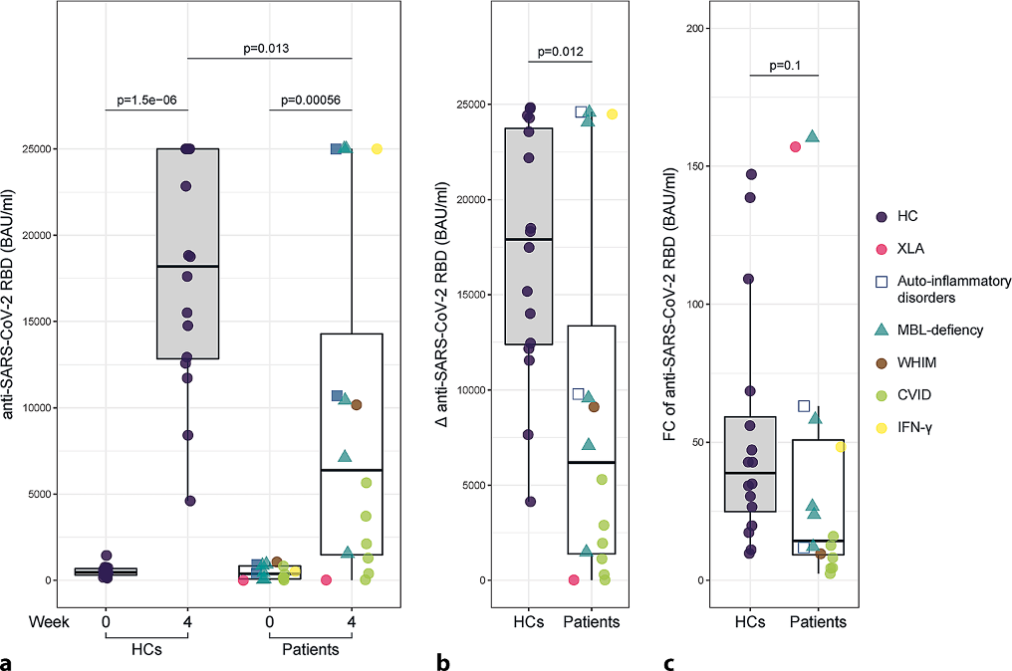
Humoral immune response at week 4 after the third vaccination. **a** Antibody levels to the receptor-binding domain (RBD) of the viral spike (S) protein in IEI/MBLdef patients (*n* = 16) and healthy controls (HCs, *n* = 16) at screening (week 0) and at week 4 after the third vaccination were determined using an anti-SARS-CoV‑2 immunoassay. **b** Absolute change (∆) of anti-RDB antibody levels between week 0 and week 4 after the third vaccination. **c** Fold change (FC) of anti-RBD antibody levels between week 0 and 4 in patients and HCs. *XLA* X-linked agammaglobulinemia, *WHIM* warts, hypogammaglobulinemia, immunodeficiency, myelokathexis, *MBL* mannose-binding lectin, *CVID* common variable immunodeficiency, *IFN*γ interferon gamma

**Fig. 2:**
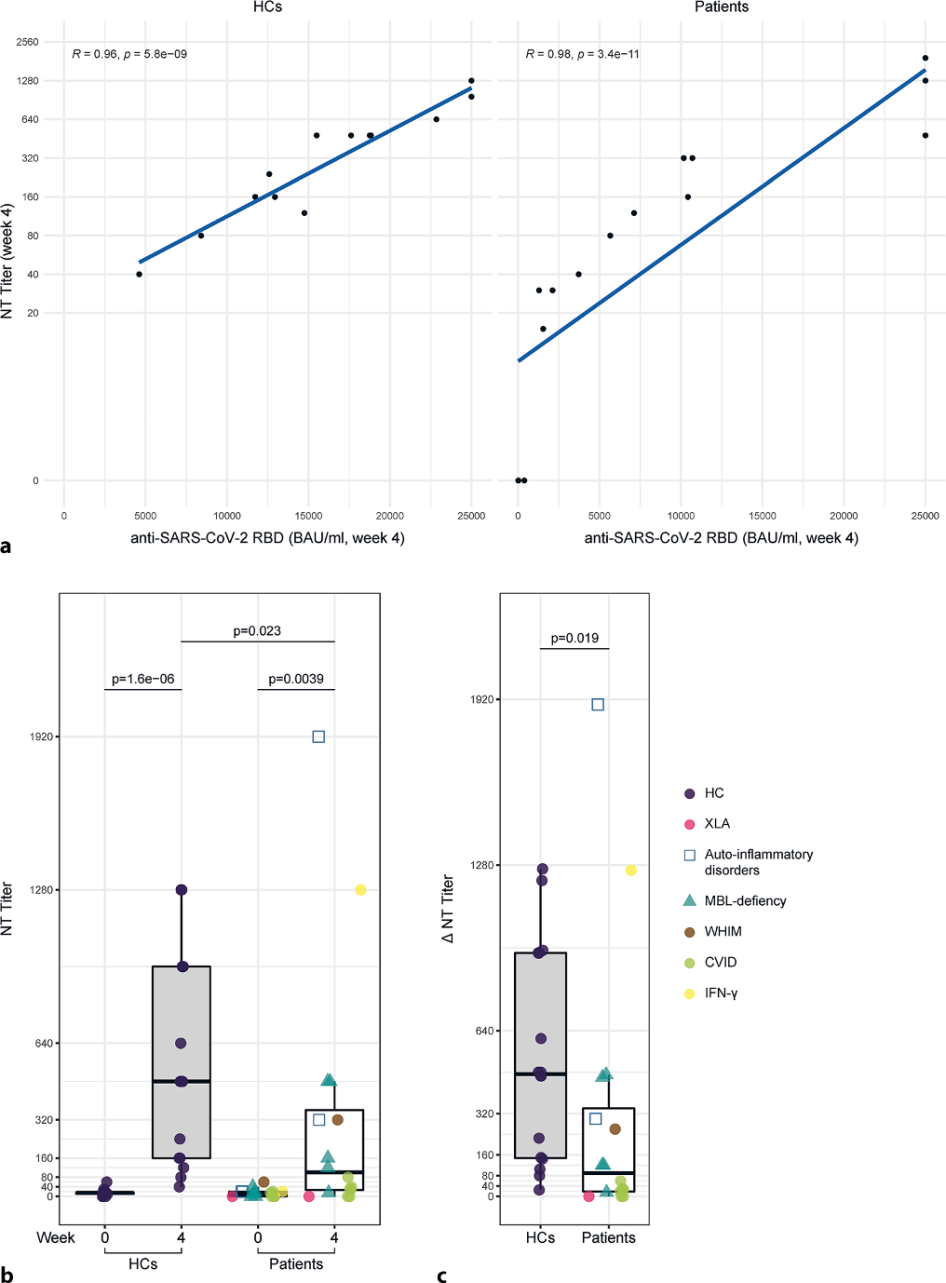
Neutralizing antibody response at week 4 after the 3rd vaccination. **a** Correlation of antibody levels to the receptor-binding domain (RBD) of the viral spike (S) protein and neutralizing antibodies (NT) against a SARS-CoV‑2 WT virus strain at week 4 after the 3rd vaccination of healthy controls (HCs, *n* = 16) and IEI/MBLdef patients (*n* = 16). **b** Neutralizing antibodies against the SARS-CoV‑2 at week 0 and week 4 after the 3rd vaccination in patients and healthy controls (HCs). **c** Absolute change (∆) of neutralizing antibodies against SARS-CoV‑2 between week 0 and week 4 after the third vaccination of HCs and patients. *XLA* X-linked agammaglobulinemia, *WHIM* warts, hypogammaglobulinemia, immunodeficiency, myelokathexis, *MBL* mannose-binding lectin, *CVID* common variable immunodeficiency, *IFN*γ interferon gamma

Anti-SARS-CoV‑2 RBD antibodies were measured at baseline (week 0) and after 4 weeks. The primary endpoint was defined as achieving antibody levels > 1500 BAU/ml. The percentage of individuals reaching this primary endpoint was lower in the patient cohort compared to HCs (IEI/MBL deficiency: 12, 75% vs. HC: 16, 100%; *p* = 0.1012). Specifically, one patient with XLA (X-linked agammaglobulinemia) and three patients with CVID (common variable immunodeficincy) did not meet the primary endpoint. At week 0 there was no significant difference in antibody levels against the SARS-CoV‑2 RBD of the spike protein between the healthy controls and the patient cohort (HC median: 455 BAU/ml, IQR 253.8–702 BAU/ml vs. IEI/MBL deficiency median: 375.5 BAU/ml, IQR 70.93–850 BAU/ml; *p* = 0.632). Of note, the interval between the second and third vaccination was significantly shorter in the patient cohort compared to the control group (*p* < 0.0001; Table 1). After vaccination, a significant increase in the production of anti-SARS-CoV‑2 RBD antibodies was detected for HC between week 0 and week 4 (week 0 median: 455 BAU/ml, IQR (303–678)) vs. week 4 median: 18,185 BAU/ml, IQR 12,853–25,000 BAU/ml; *p* = 1.5^−6^; Fig. 1a) as well as for the patient cohort (week 0: median: 375 BAU/ml, IQR 83–832 BAU/ml vs. week 4: median: 6390 BAU/ml, IQR 1481–14,275 BAU/ml, *p* = 0.00056; Fig. 1a), indicating a robust response to vaccination in both cohorts; however, the median antibody levels at week 4 were significantly lower in the IEI/MBLdef cohort (median: 6390 BAU/ml, IQR 1481–14,275) when compared to HC (median: 18,185 BAU/ml, IQR 12,853–25,000; *p* = 0.013; Fig. 1a). Following this, the absolute change of anti-SARS-CoV‑2 RBD antibodies (∆) between week 0 and week 4 was significantly lower in the IEI/MBLdef cohort (median: 6190 BAU/ml, IQR 1393–13,361) than in the HC cohort (median: 17,903 BAU/ml, IQR 12,385–23,739, *p* = 0.012; Fig. 1b). Additionally, we observed a lower, albeit not statistically significant, median fold change (FC) in anti-SARS-CoV‑2 RBD antibody levels in the patient cohort (median FC = 14.31, IQR 9.26–50.84) as compared to the healthy controls (median FC = 38.92, IQR 24.92–59.18, *p* = 0.102, Fig. 1c). Interestingly, the patient with X‑linked agammaglobulinemia (XLA) exhibited the highest fold change in antibody levels, reaching 15.7 BAU/ml after the third vaccination, which might indicate a minimal antibody production after repetitive vaccination. Furthermore, levels of anti-SARS-CoV‑2 RBD antibodies significantly correlated with titers of neutralizing antibodies against SARS-CoV‑2 (NT) at week 4 in the IEI/MBLdef cohort (R = 0.98, *p* = 3.4^−11^) as well as in the HC group (R = 0.96 *p* = 5.8^−9^), Fig. 2a. Both cohorts developed a robust increase in NT titers 4 weeks after vaccination when compared to baseline (HC: week 0 median: 15, IQR 10–16.25 vs. week 4 median: 480, IQR 160–960, *p* = 1.6^−6^; IEI/MBLdef: week 0: median: 12.5, IQR 0–20 vs. week 4 median: 100, IQR26.25–360, *p* = 0.0039; Fig. 2b). Levels of neutralizing antibodies against SARS-CoV‑2 were significantly lower in the patient group (median NT level: 100, IQR 26.25–360) when compared to HC at week 4 after vaccination (median NT level: 480, IQR 160–960; *p* = 0.023; Fig. 2b). The absolute change in the production of neutralizing antibodies between week 0 and week 4 was significantly decreased in the patient cohort (median absolute change (∆) of NT level: 90, IQR 18.75–340) in comparison to the control group (median absolute change (∆) of NT level: 472.5, IQR 148.75–940, *p* = 0.019; Fig. 2c). Overall, the humoral immune response to a third COVID-19 vaccination was markedly diminished in the patient cohort when compared to healthy controls.

### Cellular immune response

**Fig. 3:**
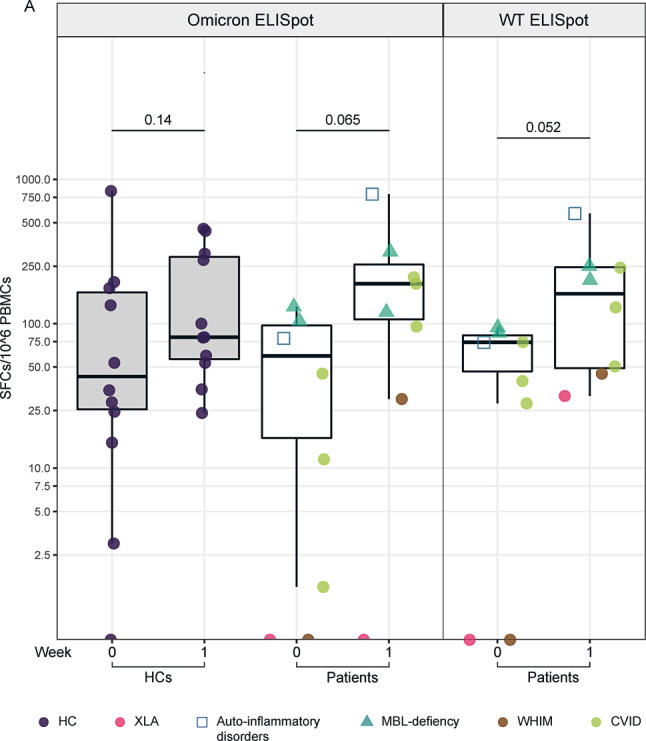
SARS-CoV-2-specific T‑cell response. SARS-CoV-2-specific T‑cell responses were determined by IFN‑γ enzyme-linked immunosorbent spot (ELISpot) assay from peripheral blood mononuclear cells (PBMC) stimulated with omicron and with wild type peptide pools (WT) before (week 0) and 1 week after the third vaccination. ELISpot results from 11 HCs and 8 patients before and 1 week after the vaccination stimulated with omicron peptides (*left panel*). Dots show the sum of total responses from S1 and S2 peptide pools (covering the S1 and S2 domain of the SARS-CoV-2 spike protein). ELISpot results from 8 patients before and 1 week after the vaccination stimulated with WT peptides (*right panel*). *XLA* X-linked agammaglobulinemia, *WHIM* warts, hypogammaglobulinemia, immunodeficiency, myelokathexis, *MBL* mannose-binding lectin, *CVID* common variable immunodeficiency, *IFN*γ interferon gamma

Before vaccination (week 0) and 1 week afterward, we conducted an ELISpot assay to evaluate the SARS-CoV-2-specific T‑cell response in 11 HC and 8 IEI/MBLdef patients. We confirmed a slight increase in SARS-CoV-2-specific spot-forming cells (SFCs) in the HC cohort between week 0 and week 1 after stimulation with omicron peptides (HC: median week 0: 34.5, IQR 19.75–155.0 vs. median week 1: 80.5, IQR 56.75–290.50 per 10^6^ SFCs, *p* = 0.14). Analysis revealed a slightly greater increase for IEI/MBLdef patients (IEI/MBLdef: median week 0: 28.25, IQR 1.125–85.25 vs. median week 1: 154.5, IQR 79.125–235.86, *p* = 0.065). In parallel, we restimulated the equivalent samples of patients with WT peptides and observed an increasing tendency of SARS-CoV-2-specific T‑cell response (IEI/MBLdef: median week 0: 57.0, IQR 21.0–77.38.0 vs. median week 1:164, IQR 49.13–245.50 per 10^6^ SFCs, *p* = 0.052, Fig. 3a), however, none of these results achieved statistical significance.

### Reactogenicity

**Fig. 4:**
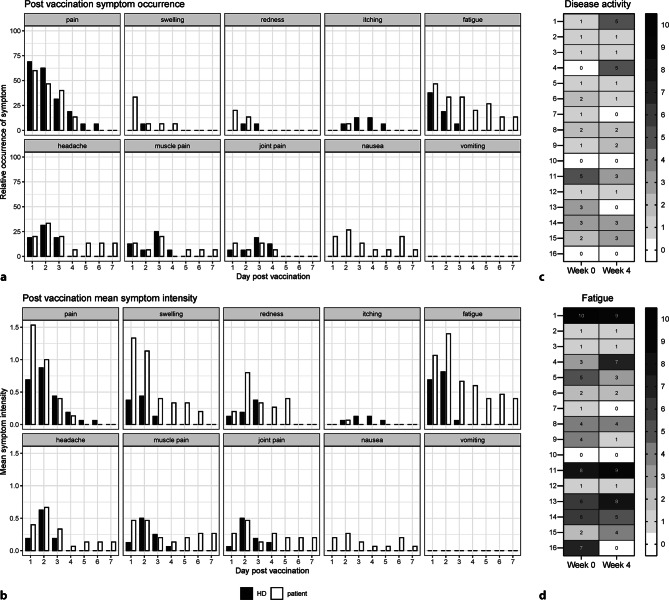
Reactogenicity after the third vaccination. Manifestations at the site of injection and systemic symptoms were recoded over a period of 7 days after vaccination. **a** Relative occurrence of the indicated symptom in IEI/MBLdef patients (*n* = 15) and healthy controls (*n* = 16). (B) Mean symptom intensity. Symptom severity was assessed on a numeric rating scale between 0 and 3. **c** and **d** Heatmap of the dynamics of the activity of the underlying disease (**c**) and fatigue (**d**) evaluated by using a numeric rating scale ranging from 0 to 10 before the 3rd vaccination and at week 4. Vertical patient numbers refer to Supplementary table 1 with patient number: 1–6 CVID (common variable immunodeficiency), 7 XLA (X-linked agammaglobulinemia), 8 WHIM (warts, hypogammaglobulinemia, immunodeficiency, myelokathexis), 9 *IFNGR1* (interferon gamma receptor 1) mutation, 10 MWS (muckle-wells-syndrome), 11 CAPS (cryopyrin-associated periodic syndrome), 12–16 MBLdef (mannose-binding lectin defficiency)

Adverse events following vaccination were systematically assessed using a paper-based diary, coupled with daily body temperature recordings. Symptoms were graded on a scale from 0 to 3, indicating absence (0) to high severity (3). All healthy controls and 15 out of 16 IEI/MBLdef patients provided diary entries. Notably, 14 out of 15 patients (93.3%) and 15 out of 16 HCs (93.8%) reported experiencing at least 1 local or systemic symptom during this monitoring period. The predominant symptom over 7 days was localized pain at the injection site (HC: 87.5%; patients: 80%), followed by fatigue (HC: 62.5%; patients: 73.3%) and headache (HC: 56.3%; patients: 53.3%). Remarkably, IEI/MBLdef patients exhibited systemic symptoms, such as headache, muscle pain and fatigue persisting for up to 7 days, whereas HCs reported shorter durations of these symptoms (Fig. 4a). Moreover, patients demonstrated a heightened severity of fatigue within the first 7 days after vaccination when compared to healthy controls, as depicted in Fig. 4b. It is noteworthy that nausea was exclusively reported in the IEI/MBLdef cohort, with 5 out of 15 patients experiencing this symptom (Fig. 4a). Conversely, no discernible disparity in body temperature was observed between patients and healthy controls, as indicated in Supplementary Fig. 1a. A comprehensive summary detailing all assessed symptoms and the frequency of occurrence is provided in Supplementary Table 2. Importantly, no occurrences of serious adverse events were documented throughout the 4‑week observation period. Before and at week 4 post-vaccination, we examined the lasting impacts on disease activity and fatigue using a PGA with a numeric rating scale from 0 to 10. Among the patients, four individuals reported an escalation in disease activity (patients 1, 4, 9 and 15). Notably, as depicted in Fig. 4c, four patients noted an amelioration in the underlying condition: patients 6, 7, 11 and 13. Additionally, 4 out of 16 patients (patients 4, 11, 13 and 15) experienced heightened fatigue at week 4 post-vaccination compared to baseline. Conversely, six patients reported a decrease in fatigue (patients 1, 5, 7, 9, 14 and 16), as shown in Fig. 4d. These findings suggest minimal to negligible prolonged effects of vaccination on the progression of underlying disease and fatigue in patients suffering from IEIs or MBL deficiency.

## Discussion

Data regarding vaccinations beyond the basic immunization in patients with inborn errors of immunity (IEIs) or MBL deficiency has been accumulating over the past few years but it still remains considerably limited in comparison to data available for patients with severe secondary immunodeficiencies [25, 26]. In the current trial, we characterized the humoral and cellular immune responses of 16 patients suffering from IEI or severe MBL deficiency compared to matched healthy controls with a limited immune response after the second dose. Our patient cohort consisted not only of patients with common variable immunodeficiency (CVID), but also of individuals with monogenetic mutations such as X‑linked agammaglobulinemia (XLA), autoinflammatory syndromes, rare mutations like a mutation in the interferon gamma receptor 1 (*IFNGR1*) gene and a patient with warts, hypogammaglobulinemia, immunodeficiency, myelokathexis (WHIM) syndrome. Of particular interest are the latter two patients, as safety data for a third vaccination in individuals with these mutations are still very rare. In alignment with previous studies in IEI patients we have demonstrated that mRNA immunization is generally immunogenic [8, 9, 12–20, 22, 27]. Notably, the magnitude of the immune response was significantly diminished in the IEI/MBLdef cohort. When comparing the fold change of anti-SARS-CoV‑2 RBD antibody and neutralizing titers, we observed significant differences between the HCs and the IEI/MBLdef group. It has been demonstrated that in immunosuppressed patients antibodies decrease over time to a greater extent than in immunocompetent subjects [9, 28]. In this study, we identified a reduction in both the fold change and absolute levels of antibodies, from week 0 to week 4 in the patient cohort. This trend persisted despite the patient group having a shorter interval between the vaccinations and might be associated with the degree of the innate immunocompromization within this cohort. An intriguing exception is observed in patients with XLA. Due to a mutation in the *Bruton tyrosine kinase* gene, the natural occurrence of an antibody response is disrupted; however, van Leeuwen et al. reported a positive antibody response to COVID-19 vaccination in some XLA patients, likely attributable to residual B cell function or B cell count, rather than contamination of anti-SARS-CoV‑2 RBD antibodies in immunoglobulin replacement therapy [16]. In our study, the XLA patient exhibited an increase in the fold change of anti-SARS-CoV‑2 RBD antibody production following the third vaccination. This patient has been receiving continuous immunoglobulin replacement therapy on a monthly basis (Supplementary Table 1). During the study period from week 0 to week 4, the patient’s immunoglobulin batch switched from privigen® (CSL Behring GmbH, Marburg, Germany) P100261870, which had been administered for 3 months, to privigen® P100313729. It is confirmed that this new batch contains SARS-CoV‑2 RBD-specific antibodies [12]. Neutralizing antibodies, which typically correlate with anti-SARS-CoV‑2 RBD antibodies, were not found in the patient’s serum. Additionally, an analysis of lymphocyte subsets revealed the complete absence of CD19 ^+^ B cells among PBMCs. Thus, in our case, the rise in anti-SARS-CoV‑2 RBD antibodies likely stems from exogenous supplementation rather than a natural immune response. Importantly, for the WHIM patient, the patient with the *IFNGR1* mutation and the patients with autoinflammatory disorders, we observed a robust immune response post-booster vaccination. The CVID is characterized by an impaired humoral immune response to polysaccharide vaccines. It has been demonstrated that the immune response of CVID patients to COVID-19 mRNA vaccines is very heterogeneous [12, 17, 19, 20] most likely due to variable B cell compartments, which is in line with our results. It has recently been speculated that the presence of CVID-associated autoimmune complications in patients is linked to a poorer antibody response following COVID-19 vaccination, keeping in mind that this subpopulation of CVID patients is more often on immunosuppressive treatment which has the potential to dampen the immune response [16]. In our CVID cohort, the patient who displayed the lowest anti-SARS-CoV‑2 RBD antibody levels after vaccination (12–31 BAU/ml), had started with rituximab treatment. This treatment was initiated between the second and third COVID-19 vaccination because of a granulomatous lymphocytic interstitial lung disease (GLILD).

The MBL is a pattern recognition molecule and its binding to mannose or sugar motifs on various antigens activates the complement system. Data about the impact of MBL on the immunogenicity of vaccines are scarce. It is hypothesized that a typical vaccine response is the most probable outcome. Consistent with this hypothesis, patients deficient in mannose-binding lectin (MBL) exhibited a robust immune response following the third vaccination, despite four out of five patients completely lacking MBL.

Patients with primary or secondary defects in the humoral immune response are capable of developing an antigen-specific cellular response. In this study, we observed a modest enhancement in the cellular immune response following restimulation with wild-type (WT) and omicron peptides. These findings underline the potential benefit of a third vaccination, even for patients with compromised humoral immunity.

There is significant hesitancy toward COVID-19 vaccination among patients with immunodeficiencies. The predominant reason for this hesitancy is uncertainty regarding the immune response and its potential impact on their underlying condition [10]. This skepticism is evident in the limited adherence to booster vaccinations among patients with IEI [19]. No serious adverse events related to the third dose were observed during the study period. The prevalence of side effects was similar between the patient group and healthy controls with the exception of nausea, which was reported exclusively by three MBL-deficient patients, one CAPS (cryopyrin-associated periodic syndrome) patient, and one CVID patient within the first 7 days. These results support the previously published good vaccine safety in patients with IEI [8, 11, 29].

Acknowledging the concerns of patients regarding the potential impact on their underlying disease, we evaluated disease activity and fatigue using a PGA. In comparison from week 0 to week 4, four patients noted an increase in disease activity, while four patients reported a decrease. Consequently, although our trial was not powered for this endpoint, we did not observe a definitive trend indicating exacerbation of the underlying immune defect. These results were even more pronounced when we assessed the manifestation of fatigue, where more patients reported a decrease in fatigue than an increase. The provided data are of high value for IEI patients and should be communicated to healthcare providers.

We acknowledge the limitations of this study. The number of subjects is very small, primarily due to the gradual reduction in the IEI/MBLdef cohort, which met the criteria for a third vaccination over time. Patients with a history of a SARS-CoV‑2 infection and those with anti-SARS-CoV‑2 RBD antibodies exceeding > 1500 BAU/ml were excluded from participation. Moreover, some patients declined or deferred additional booster doses following the initial vaccination. Certainly, larger cohorts are imperative to comprehensively investigate the immunogenicity and efficacy of COVID-19 vaccination in patients with IEI and MBL deficiency. Additionally, the relatively short observation period underlines the necessity for prolonged surveillance to evaluate the potential for long-term effects.

In summary, our data substantiate the safety and immunogenicity of repetitive COVID-19 vaccinations in patients with inborn errors of immunity and MBL deficiency. This strategy is especially advisable for individuals demonstrating an initial suboptimal response to vaccination. Information on vaccination response and safety is particularly important for this vulnerable group of patients and our data will provide future guidance for any upcoming vaccination strategies.

## Supplementary Information

Supplementary Table 1: demographic information, presentation of the immunodeficiency, treatment and vaccination details; supplementary Table 2: patient reported reactogenicity during the first week post vaccination; supplementary Figure 1: body temperature post vaccination
