# Supplementary material for: Humoral and cellular response to the third COVID-19 vaccination in patients with inborn errors of immunity or mannose-binding lectin deficiency: A prospective controlled open-label trial
Source: Wien Klin Wochenschr. 2024 Oct 24;136(21-22):598–607. doi: 10.1007/s00508-024-02459-6 (PMC11535081; doi:10.1007/s00508-024-02459-6)
Supplement: Supplementary file 1 — Supplementary Table 1: demographic information, presentation of the immunodeficiency, treatment and vaccination details; supplementary Table 2: patient reported reactogenicity during the first week post vaccination; supplementary Figure 1: body temperature post vaccination [file 508_2024_2459_MOESM1_ESM.docx]

Supplementary material

**Supplementary Table 1**

| **Patient no.** | **Age (y)** | **Gender** | **Underlying diagnosis** | **Diagnostic criteria** | **Autoimmune phenomena during the course of disease** | **Infectious complications during the course of disease** | **IgRT (g/month)** | **Trade name of IgRT** | **Immunomodulator/antibiotic** | **Primary immunization** | **Booster** |
| --- | --- | --- | --- | --- | --- | --- | --- | --- | --- | --- | --- |
| 1 | 56 | F | CVID | IgG: 527 | arthralgia, arthritis, fatigue, myalgia, splenomegaly, IBD-like | chronic gastritis | no |  | - | BNT162b2 | BNT162b2 |
|  |  |  |  | IgA: 89.5 |  |  |  |  |  |  |  |
|  |  |  |  | IgM: < 5.2 |  |  |  |  |  |  |  |
| 2 | 41 | F | CVID | IgG: < 195 | SLE-like, fatigue, leucopenia, lymphadenopathy, hepatomegaly, splenomegaly, history of CNS vasculitis | recurrent cystitis | 24 | Hizentra® | Hydroxychloroquine | BNT162b2 | BNT162b2 |
|  |  |  |  | IgA: < 6.3 |  |  |  |  |  |  |  |
|  |  |  |  |  |  |  |  |  |  |  |  |
|  |  |  |  |  |  |  |  |  |  |  |  |
| 3 | 46 | F | CVID | IgG: < 195 | none | recurrent pneumonia | 20 | privigen® | - | BNT162b2 | BNT162b2 |
|  |  |  |  | IgA: < 7.2 |  |  |  |  |  |  |  |
| 4 | 29 | F | CVID | IgG: 206, IgA: <7.0 | arthralgia, arthritis, fatigue, GLILD, hepatomegaly , IBD-like, lymphadenopathy, leucopenia, splenomegaly, thrombocytopenia, | pneumonia, recurrent cystitis, pulmonary aspergillosis | 48 | Hizentra® | Rituximab | BNT162b2 | BNT162b2 |
| 5 | 53 | M | CVID | IgG: 286 | fatigue, IBD-like | pneumonia | 40 | gammanorm® | - | BNT162b2 | BNT162b2 |
|  |  |  |  | IgA: < 6.8 |  |  |  |  |  |  |  |
| 6 | 60 | M | CVID | IgG: < 195 | arthralgia | bronchitis, sinusitis | 20 | privigen® | - | BNT162b2 | BNT162b2 |
|  |  |  |  | IgA: < 33 |  |  |  |  |  |  |  |
| 7 | 38 | M | XLA | genetic | none | chronic sinusitis , chronic otitis | 16 | privigen® | - | mRNA-1273 | mRNA-1273 |
| 8 | 26 | F | WHIM | genetic | leucopenia, splenomegaly | recurrent pneumonia, bronchitis, otitis, warts/HPV | 16 | Hizentra® | - | BNT162b2 | BNT162b2 |
| 9 | 39 | F | Mutation in IfngR1 | genetic | rheumatoid arthritis, fatigue | recurrent pneumonia, bronchitis, otitis, chronic infection with M.avium (lung and CNS) | no |  | Salazopyrin, Dexamethason, Clarithromycin, Rifampicin, Tezdizolid, Moxifloxacin | BNT162b2 | BNT162b2 |
| 10 | 40 | M | Muckle-Wells syndrome (MWS) | genetic | arthralgia | none | no |  | Hydroxychloroquine | ChAdOx1 | BNT162b2 |
| 11 | 42 | F | CAPS | genetic diagnosis by third party | arthralgia, fatigue, fever, myalgia, pleurisy, rash, lymphadeno-pathy, IBD-like, aseptic meningitis | recurrent oral ulcers, chronic gastritis, recurrent pyelonephritis | no |  | Canakinumab | BNT162b2 | BNT162b2 |
| 12 | 52 | F | MBL-deficiency and selective deficiency in pneumococcal antibody response | pneumococcal 1:128, no dynamic after vaccination, MBL 16.3 | IBD-like, bronchiectasis | bronchitis, sinusitis, chronic gastritis | no |  | - | BNT162b2 | BNT162b2 |
| 13 | 25 | F | MBL-deficiency | MBL <0.5 | arthralgia, myalgia, lymphadeno-pathy | recurrent oral ulcers | no |  | - | BNT162b2 | BNT162b2 |
| 14 | 41 | F | MBL-deficiency | MBL <0.5 | fatigue, rash, Raynaud´s phenomenon, lymphadeno-pathy, IBD-like | recurrent pneumonia , sinusitis , recurrent oral ulcers, chronic gastritis | no |  | - | ChAdOx1 | BNT162b2 |
| 15 | 39 | F | MBL-deficiency | MBL <0.5 | fatigue, myalgia, rash, IBD-like | chronic gastritis, recurrent herpes simplex infection, recurrent cystitis, recurring abscesses | no |  | - | BNT162b2 | mRNA-1273 |
| 16 | 69 | F | MBL-deficiency | MBL <0.5 | none | bronchitis, sinusitis, recurrent cystitis | no |  | - | BNT162b2 | mRNA-1273 |

Supplementary Table 1: The column “diagnostic criteria” shows the markers of immunodeficiency at the time of initial diagnosis. F, female; M, male; IgRT (g/month), Immunoglobulin replacement therapy in gram per month. IgG/IgM/IgA values are displayed in mg/dl. Cell counts are expressed in absolute numbers per microliter (cell/µl) and MBL levels in nanogram per microliter (ng/ml). The cutoffs/normal ranges are the following: IgG: 700-1600 mg/dL, IgG1: 280-800 mg/dL, IgG2: 169-786 mg/dL, IgA 70-400 mg/dL, IgM: 40-230 mg/dL, MBL: >300 ng/ml, CD19^+^: 100-500 c/µl; IgD^+^ CD27^+^ non–class-switched memory B cells: 10-110 c/µl; IgD^-^ CD27^+^ class-switched memory B cells: 10-80 c/µl, anti pneumococcal IgG 1:200.

**Supplementary Table 2**

| symptom reported during the first week post vaccination | patient n=15 | HC n=16 |
| --- | --- | --- |
| any symptom (%) | 14 (93,3) | 15 (93,8) |
| pain at vaccination site (%) | 13 (86,7) | 14 (87,5) |
| fatigue (%) | 11 (73,3) | 10 (62,5) |
| muscle pain (%) | 5 (33,3) | 5 (31,3) |
| headache (%) | 8 (53,3) | 9 (56,3) |
| joint pain (%) | 5 (33,3) | 4 (25) |
| redness at vaccination site (%) | 6 (40) | 3 (18,8) |
| swelling at vaccination site (%) | 7 (46,7) | 4 (25) |
| itching at vaccination site (%) | 1 (6,7) | 2 (12,5) |
| nausea (%) | 5 (33,3) | 0 (0) |
| vomiting (%) | 0 (0) | 0 (0) |

Supplementary Table 2: Reported Reactogenicity. Symptoms recorded in a standardized format (NRS 0-3) for 7 days.

**Supplementary Figure 1**


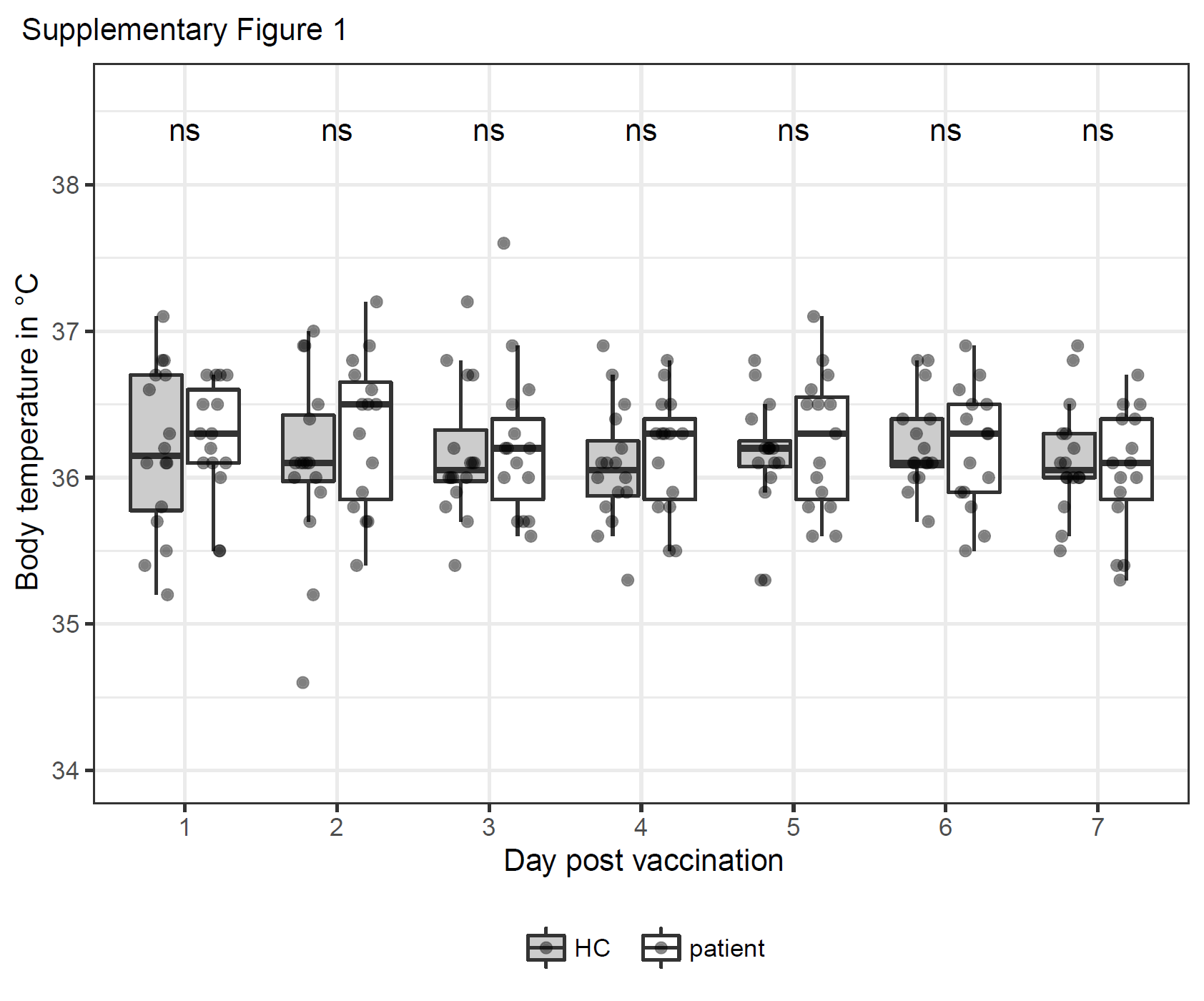


Supplementary Figure 1: Body temperature at the same time of day over a period of 7 days post booster vaccination. HC; healthy control;
